# Surgical Complications After Reverse Total Shoulder Arthroplasty and Total Shoulder Arthroplasty in the United States

**DOI:** 10.5435/JAAOSGlobal-D-21-00146

**Published:** 2021-07-20

**Authors:** Gabrielle C. Ma, Kendall E. Bradley, Hayley Jansson, Brian T. Feeley, Alan L. Zhang, C. Benjamin Ma

**Affiliations:** From the Department of Orthopaedic Surgery, University of California, San Francisco, CA.

## Abstract

Shoulder arthroplasty has become popular in the treatment of degenerative shoulder conditions in the United States. Shoulder arthroplasty usage has expanded to younger patients with increased surgical indications.

**Methods:**

Reverse total shoulder arthroplasty (RTSA) and TSA patient records with the 1-year follow-up between 2015 and 2018 were queried from the nationwide PearlDiver Mariner Shoulder Database using International Classification of Disease-10 codes. Chi-square analysis was done to compare the demographics, surgical complications, and revision procedures between RTSA and TSA.

**Results:**

From 2010 to 2018, there was an increase in shoulder arthroplasty cases because of RTSA. The overall surgical complication and revision procedure rates were 2.26% and 3.56% for RTSA, and 6.36% and 2.42% for TSA. Patients older than 50 years had statistically lower surgical complications after RTSA than TSA (2.25% versus 3.94%, *P* < 0.05), whereas no statistical difference between RTSA and TSA for patients younger than 50 years (10.06% versus 7.45%, *P* = 0.19). Male patients had higher RTSA complication rates (3.12% versus 2.28%, *P* < 0.05), whereas female patients had higher TSA (4.86% versus 5.92%, *P* < 0.05). History of tobacco, depression, and obesity were risk factors for higher complications.

**Conclusion:**

RTSA has become more commonly done than TSA in the United States. Older patients who underwent shoulder arthroplasty had lower surgical complication. TSA had a higher surgical complication rate than RTSA for patients older than 50 years.

There has been a steady increase in the number of shoulder arthroplasty procedures being done in the United States, and the volume is projected to continue to increase dramatically in the next 5 years.^1^ Although anatomic shoulder arthroplasties have been a mainstay in the treatment of shoulder arthritis, there has been an increase in reverse total shoulder arthroplasty (RTSA) by almost 200% over the past decade.^1,2^ Studies have also reported that shoulder arthroplasty procedures are done on younger patients and expanded surgical indications.^3^

Complications from anatomic total shoulder arthroplasty (TSA) are approximately 10% and include hardware loosening, glenoid wear, instability, and rotator cuff tear.^4,5^ The historic complication rate of RTSA ranges from 10% to 47%.^4,6,7,8,9,10,11^ These complications include instability, periprosthetic fracture, infection, and component loosening.^4^ When comparing modern shoulder arthroplasty implants in the United States, some have revealed that there are similar outcomes and some have shown differences in relation to patient-reported outcomes, range of motion, complication rate, and revision procedures.^12,13^ Despite elderly patients often having more medical comorbidities, previous studies have noted that complications are higher in patients younger than 65 years for both RTSA and TSA.^14^ Patients older than 80 years are reported to have a surgical complication rate of less than 15%, even for revision surgeries.^15^

Database studies are a powerful means to look at broad trends and synthesis of data.^16,17,18,19,20,21^ With the introduction of International Classification of Diseases (ICD)-10 in 2015, the ability to track complications based on laterality has allowed for more precise examination of surgical outcomes.^22^ Notably, this study occurs over a relatively short period because of ICD-10 laterality. As a result, patients with minimum 1 year and maximum 3 year active are analyzed. In this study, our goal was to use a national database to look at the surgical complication rates after shoulder arthroplasty using ICD-10 codes. Our hypothesis was that the complication rate will be lower in older patients and that the complication rate will be higher in RTSA when compared with TSA.

## Methods

A retrospective search of patients who had undergone RTSA and TSA was done using the nationwide PearlDiver Mariner Shoulder Database. Using ICD-9 and ICD-10 codes, RTSA and TSA occurrences were found between 2010 and 2018 to see the incidence for both shoulder arthroplasty procedures. For our study cohort, ICD-10 codes were used to specify laterality and search for patient records between 2015 and 2018. Only those with at least 1-year follow-up were included in the study cohort. The ICD-10 codes for RTSA (0RRK00Z, 0RRJ00Z) and TSA (0RRK0JZ, 0RRJ0JZ) were searched for 1-year active patients. Demographic data such as age and sex were collected. Patients were stratified into designated age groups of younger than 50, 50 to 60, and 60 to 75 years, and older than 75 years. Comorbidities such as tobacco usage, depression, and obesity were also gathered.

Complications were classified by ICD-10 diagnostic and procedure codes. Surgical complications included infection and drainage, dislocation, loosening, fracture, and rotator cuff tear (only for TSA) (Appendix Table 1, http://links.lww.com/JG9/A148). Procedure codes included irrigation and débridement, explant and insertion of spacer, reduction of dislocated prosthesis, revision arthroplasty, and rotator cuff repair (only for TSA) (Appendix Table 1, http://links.lww.com/JG9/A148). Surgical complications and revision procedures for each age range were found within 1 year after RTSA and TSA procedures. Laterality was tracked throughout the analysis using ICD-10 codes.

Statistical analysis was done using the R software program integrated into PearlDiver. Chi-square analysis was done for the demographic and quantitative clinical characteristics. Comparisons were made between RTSA and TSA and between different age ranges. Significance was defined as *P* < 0.05.

## Results

**Figure 1:**
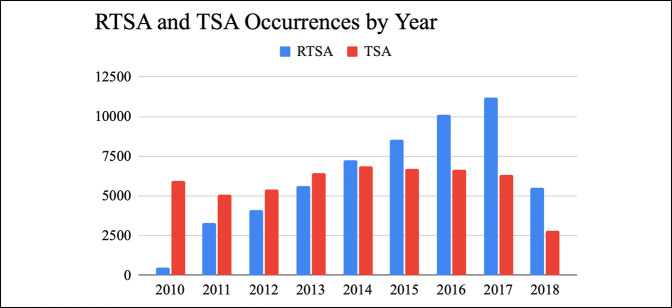
Bar diagram showing the shoulder arthroplasty trend over time shown by the number of RTSA and TSA occurrences by year. RTSA = reverse total shoulder arthroplasty

**Figure 2:**
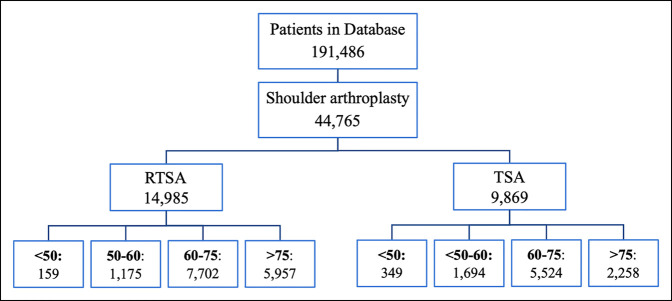
Figural representation showing the 1-year follow-up populations from the 2015-2018 in the PearlDiver Mariner Shoulder Database. Shown is the distribution of patients who had a shoulder reverse and total arthroplasty for each age group.

From 2010 to 2018, there was a steady increase in the total number of shoulder arthroplasties being done in the United States. TSA initially had a higher number of occurrences than RTSA; however, RTSA has increased and surpassed the number of TSA occurrences since 2014 (Figure 1). In 2017, there were 11,187 RTSA and 6,316 TSA being done. The incidence of RTSA has steadily increased throughout the year, whereas the incidence of TSA has remained similar. In the study cohort where we looked at complications using ICD-10 codes, there were a total of 44,765 patients who had undergone shoulder arthroplasty from 2015 to 2018: 28,750 RTSA patients and 17,252 TSA patients. Overall, 14,985 RTSA patients (52.12%) and 9,869 TSA patients (57.2%) with at least 1-year follow-up were included in the database (Figure 2). Female patients had higher percentage of RTSA and TSA procedures than male patients (63.2% versus 36.8% and 51.8% versus 48.2%, respectively; *P* < 0.05) (Table 1).

**Table 1:** Demographics of Sex, Tobacco Usage, Depression, and Obesity for the 1-Year Follow-up Base Population

Demographics	RTSA, n = 14,985		*P*	TSA, n = 9,869		*P*
Sex (female)	63.2%	9,470	<0.05	51.8%	5,115	<0.05
Sex (male)	36.8%	5,515		48.2%	4,754	
History of tobacco usage	29.8%	4,460	<0.05	29.5%	2,913	<0.05
No history of tobacco usage	70.2%	10,525		70.5%	6,956	
Depression	46.7%	6,992	<0.05	43.5%	4,295	<0.05
No depression	53.3%	7,993		56.5%	5,574	
Obesity	43.6%	6,532	<0.05	47.5%	4,692	<0.05
No obesity	56.4%	8,453		52.5%	5,177	

RTSA = reverse total shoulder arthroplasty

Chi-square analysis within RTSA and TSA demographics with significance defined as <0.05.

**Figure 3:**
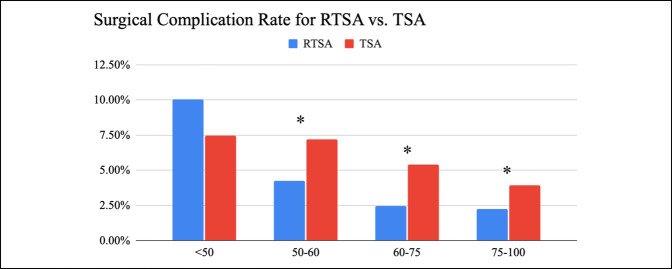
Bar diagram showing the rate of surgical complications for RTSA and TSA by the age group with the 1-year follow-up (<50, 50 to 60, 60 to 75, and >75). Chi-square analysis between RTSA and TSA for each age group. Significance defined as <0.05 and indicated by asterisk. RTSA = reverse total shoulder arthroplasty

**Figure 4:**
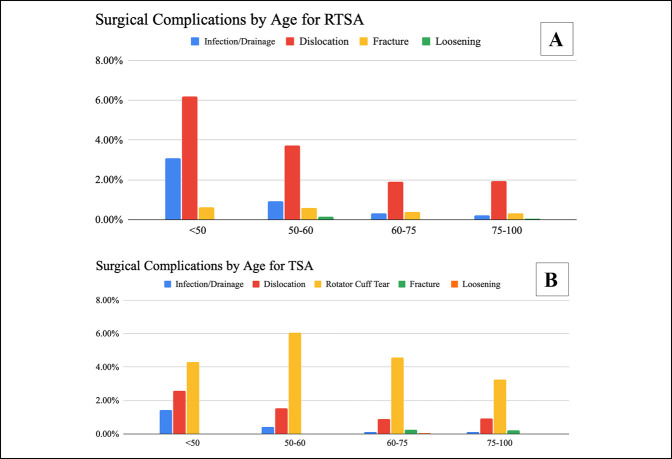
**A**, **B**, Bar diagram showing the age breakdown by the type of surgical complication after RTSA (**A**) and TSA (**B**). Surgical complications included infection and drainage, dislocation, fracture, loosening, and rotator cuff tear for TSA. RTSA = reverse total shoulder arthroplasty

Both RTSA and TSA had lower surgical complication rates with increasing age. After RTSA, there were 338 diagnostic surgical complications (2.26%). There were 534 diagnostic surgical complications after TSA (5.4%). RTSA had statistically lower complication rates when compared with TSA for all age groups with patients older than 50 years (2.25% versus 3.94%, *P* < 0.05); there was no statistically significant difference found between RTSA and TSA surgical complications in patients younger than 50 years (10.06% versus 7.45%, *P* = 0.19). Patients older than 75 years had the lowest surgical complication rate for both RTSA and TSA (2.25% and 3.94%, *P* < 0.05) (Figure 3). The most common surgical complication after RTSA was dislocation and for TSA was rotator cuff tear (Figure 4).

Statistically significant differences in the surgical complication rates between the sex and the type of shoulder arthroplasty are listed in Table 2. After RTSA, male patients were more likely to have complications (3.12% versus 2.28%, *P* < 0.05) and revision procedures (5.80% versus 3.25%, *P* < 0.05) than female patients. Conversely, after TSA, female patients were more likely to have complications and revision procedures done when compared with male patients (5.92% versus 4.86% and 2.83% versus 2.12%, respectively; *P* < 0.05). History of tobacco usage, depression, and obesity all showed increased complications and revision procedures after both RTSA and TSA (Table 1).

**Table 2:** Demographics of Sex, Tobacco Usage, Depression, and Obesity for Diagnostic Complication and Revision Procedure Populations

Demographics	Diagnostic Complications						Revision Procedures					
	RTSA, n = 338		*P*	TSA, n = 534		*P*	RTSA, n = 628		*P*	TSA, n = 239		*P*
Sex (female)	2.28%	216	<0.05	5.92%	303	<0.05	3.25%	308	<0.05	2.83%	141	<0.05
Sex (male)	3.12%	172		4.86%	231		5.80%	320		2.12%	98	
History of tobacco usage	3.65%	163	<0.05	6.39%	186	<0.05	5.45%	243	<0.05	2.80%	79	0.11
No history of tobacco usage	2.14%	225		5.00%	348		3.66%	385		2.30%	160	
Depression	3.26%	228	<0.05	6.73%	289	<0.05	4.83%	338	<0.05	3.51%	146	<0.05
No depression	2.00%	160		4.40%	245		3.63%	390		1.67%	93	
Obesity	3.02%	197	<0.05	5.69%	267	0.13	5.13%	335	<0.05	2.75%	125	0.14
No obesity	2.26%	191		5.16%	267		3.47%	293		2.20%	114	

RTSA = reverse total shoulder arthroplasty

Chi-square analysis between and within RTSA and TSA demographics for complication and procedures with significance defined as <0.05.

**Figure 5:**
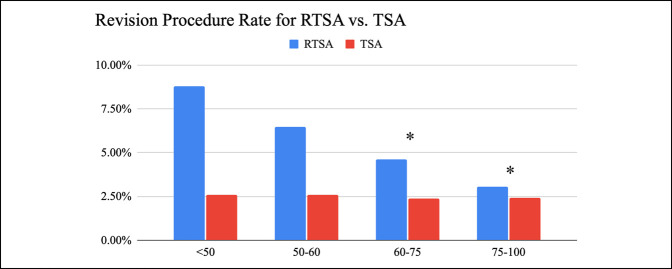
Bar diagram showing the rate of revision procedures for RTSA and TSA by the age group with the 1-year follow-up (<50, 50 to 60, 60 to 75, and >75). Chi-square analysis between RTSA and TSA for each age group. Significance defined as <0.05 and indicated by asterisk. RTSA = reverse total shoulder arthroplasty

A total of 628 revision procedures after RTSA and 239 revision procedures after TSA were found. The rates decreased throughout all age groups with patients older than 75 years having the lowest rate of revisions (3.06% versus 2.44%, *P* < 0.05). After RTSA, the rates of revision procedures were found to be higher among all age groups (Figure 5). Revision procedures were consistent throughout all age groups after TSA (Figure 4). Of all of the procedure types, revision shoulder arthroplasty was the most common procedure done (Tables 3 and 4).

**Table 3:** Revision Procedure Breakdown by Type After RTSA

Age	Irrigation and Débridement	Removal	Relocation	Revision
<50	0.62%	0.62%	0.00%	3.70%
50-60	0.17%	0.17%	0.17%	4.32%
60-75	0.36%	0.36%	0.08%	3.43%
75-100	0.25%	0.25%	0.18%	2.18%

RTSA = reverse total shoulder arthroplasty

**Table 4:** Revision Procedure Breakdown by Type After TSA

Age	Irrigation and Débridement	Removal	Relocation	Rotator Cuff Repair	Revision
<50	0.29%	0.29%	0.00%	0.00%	1.15%
50-60	0.18%	0.18%	0.00%	0.06%	2.00%
60-75	0.14%	0.14%	0.00%	0.05%	1.82%
75-100	0.13%	0.13%	0.04%	0.00%	3.17%

RTSA = reverse total shoulder arthroplasty

## Discussion

Shoulder arthroplasty has continued to increase in the United States.^2^ Our study showed a steady increase of RTSA during the entire study period with the incidence of TSA remaining the same. In a previous single-center study, RTSA was reported to be the most common primary shoulder arthroplasty in 2015.^2^ The authors found that a number of RTSAs done for osteoarthritis and irreparable rotator cuff tears have increased, and the proportion of RTSAs done for rotator cuff tear arthropathy have decreased. They also found that the mean age for shoulder arthroplasty has decreased.^24^ Another study using the Nationwide Inpatient Sample reported the TSA medical complication rate to be 6.7%. Our more recent analysis indicated lower TSA and RTSA surgical complications compared with previous literature, which indicates an improvement in procedures and outcomes.^4^

We have proved that our hypothesis that complication rates are lower in older patients in the United States. The higher complication rate in younger patients may reflect a greater complexity of shoulder conditions that require replacement. Younger patients may also have higher functional demand after the procedure. Despite previous studies noting high complication rates in RTSA, we did not find RTSA to have statistically higher complications when compared with TSA in patients younger than 50 years.^4,11^ However, TSA was found to have statistically higher complication rates when compared with RTSA for patients older than 50 years. A previous national TSA study indicated higher medical complications after surgery for older patients as well.^23^ This may be a result of the types of complications that TSA patients experience in the first year. Rotator cuff tear was the most common complication after TSA, whereas dislocation was the most common complication after RTSA. RTSA is commonly done for shoulders with dysfunctional rotator cuff tendons which is prevalent for older patients. This may be an important consideration when counseling older patients on the risks and benefits of TSA versus RTSA.

Besides age, we also found that history of tobacco usage, depression, and obesity has increased complications. These are common comorbidities that have been shown to affect outcomes after different musculoskeletal procedures.^24,25,26,27^ Some of these comorbidities are modifiable and should be counseled with patients before elective surgical procedures. In addition, a study done on a Nationwide Inpatient database focused on TSA procedures from 2006 to 2010 and found medical complications of 6.7% patients.^23^ Previous studies have shown that there can be gender differences in expectation of outcomes after shoulder arthroplasty, resulting in different patient-reported shoulder outcome scores despite similar pain scores and range of motion.^29,30^ In this study, we also found that male patients have more complications and revision procedures after RTSA, whereas female patients were more likely to have complications and revision procedures after TSA. Considering that these differences were quite small, additional evaluation is needed to determine its significance in clinical application.

Despite the focus on complications and historic data, revision procedures are rare for both procedures. Urgent need for revision procedures, such as infection, dislocation, and fracture, is uncommon for both procedures in the first year. Patients who underwent TSA have a higher complication rate than RTSA for the older age group, but the contrary was found for revision procedures. The low revision rate may be related to the patients' tolerance of rotator cuff tears after arthroplasty or preserved function of the shoulder, which is not being measured in this study. Notably, not all surgical diagnostic complications were followed by revision procedures.

Several limitations exist for this study. A low rate of follow-up was observed at 1 year, less than 60% for both TSA and RTSA. This is a limitation for all database studies when inclusion is dependent on the patient's insurance enrollment. Long-term follow-up can be difficult in these database analyses when patients may change their insurance enrollment during the study period. Our study focused only on the surgical complication rates and did not include other medical complications. Medical complications are higher for older patients, but our goal for this analysis was to focus on surgical complications and subsequent procedures.^30^ The transition from ICD-9 to ICD-10 has also brought some challenges. Clinicians have needed to adjust to the new coding system, which may have had an influence on the code accuracy used in this study. However, it would have been difficult to assure that subsequent procedures were done on the same joint as the index procedure prior the introduction of ICD-10 codes. There is also the possibility that codes used to define complications may have been confounded by inappropriate coding by providers. In addition, patient population is limited to at most 3-year follow-up because of the introduction of ICD-10 in 2015. Additional evaluation of complications over a longer period of time will be beneficial in understanding RTSA and TSA procedures in the United States. Despite these limitations, the use of ICD-10 codes and the specification of laterality do provide a more detailed tracking of diagnosis and procedures for a large nationwide sample.

## Conclusion

The results of our national study demonstrated that RTSA has become more commonly done than TSA, and the increase in RTSA has contributed to the increased total number of shoulder arthroplasties done in the United States. Older patients who underwent shoulder arthroplasty had lower surgical complication rates when compared with younger patients. Specifically, RTSA was found to have lower complication rates than TSA for patients older than 50 years. No statistically significant difference was observed in the complication rates between RTSA and TSA for patients younger than 50 years. The most common surgical complication for RTSA was dislocation and for TSA was rotator cuff tear.
